# Optimization of Microwave-Assisted Fracture Energy Recovery in Early-Damaged Asphalt Mixtures: Damage-State Regulation by Basalt Fiber Reinforcement

**DOI:** 10.3390/ma19163536

**Published:** 2026-08-20

**Authors:** Bo Li, Jian Hu, Yu Wang, Aihong Kang, Zhengguang Wu

**Affiliations:** 1 College of Civil Engineering and Transportation, Yangzhou University, Yangzhou 225127, China; libo@yzu.edu.cn (B.L.); mz120241075@stu.yzu.edu.cn (J.H.); kahyzu@163.com (A.K.); zgwu@yzu.edu.cn (Z.W.); 2 Department of Civil Engineering, University of Ottawa, Ottawa, ON K1N5Y1, Canada

**Keywords:** early fracture damage, basalt fiber reinforcement, microwave-assisted recovery, damage-state regulation, orthogonal design, asphalt mixture

## Abstract

Microwave-assisted recovery provides a potential approach for restoring fracture damage in asphalt mixtures, but previous studies have mainly focused on heating and curing conditions, while the role of the pre-heating fracture state remains less understood. This study investigated microwave-assisted fracture energy recovery from a damage-state regulation perspective by comparing a control asphalt mixture (CAM) with a basalt fiber-reinforced asphalt mixture (BFAM). Semi-circular bending (SCB) tests were combined with an L9 orthogonal design to evaluate three pre-heating conditions, target surface temperatures of 45–85 °C, and curing times of 6–24 h. Rather than directly enhancing binder recovery, basalt fiber reinforcement increased the initial fracture resistance and altered the relative fracture condition reached under a given external load. The recovery index *RI* ranged from 20.7% to 55.9% for CAM and from 35.2% to 82.3% for BFAM. Main-effects ANOVA showed that the pre-heating damage condition had the largest main-effect contribution within the adopted L9 framework, reaching 82.3% for CAM and 95.2% for BFAM, substantially exceeding those of target surface temperature and curing time. Under a comparable external load of approximately 2.5 kN, CAM reached the 70% *P_max_* condition, whereas BFAM remained at the 40% *P_max_* condition, with corresponding mean *RI* values of 42.8% and 76.9%. These results support a proposed conceptual damage-state regulation framework within the investigated material and experimental conditions, in which basalt fiber reinforcement preserves a more favorable pre-heating state and thereby greater recovery potential. The findings highlight the importance of improving fracture resistance and applying microwave-assisted treatment before extensive fracture development occurs, while broader validation is required before generalizing the proposed framework to other materials or field conditions.

## 1. Introduction

Asphalt mixtures are progressively damaged by repeated traffic loading, temperature variation, moisture ingress and oxidative aging during service. These actions initiate microcracks and promote their growth into fracture-related distresses, such as fatigue cracking and low-temperature cracking. At the early stage of damage, however, the crack interface may still retain partial contact and material continuity, which provides greater potential for recovery before severe crack opening occurs. Once the crack interface becomes widely separated or the aggregate–binder bond is substantially weakened, the possibility of restoring fracture-related mechanical capacity is considerably reduced. Therefore, the recovery of asphalt mixtures should not be interpreted only as a material healing process after complete fracture, but also as a damage-state-dependent process governed by the condition of the crack interface before treatment [1,2,3,4]. In this study, this dependence is described using a proposed “recoverable damage window” framework, referring to the range of pre-heating fracture states within which meaningful fracture energy recovery can still be achieved. This term is introduced here as a conceptual framework rather than as an established damage parameter.

Microwave-assisted recovery has attracted attention because microwave energy can rapidly heat asphalt mixtures and activate the binder near damaged interfaces. The resulting temperature rise reduces binder viscosity and promotes binder flow, wetting, diffusion and interfacial re-bonding, which may partially restore fracture energy after damage [5,6,7,8,9,10,11]. Previous studies have demonstrated the feasibility of microwave- or induction-assisted healing using bending, fracture and fracture–healing–fracture procedures, and have shown that the recovery level is affected by the thermal condition and the post-heating rest period [5,10,11]. However, these studies have generally emphasized whether thermal activation can produce recovery and how heating conditions or material modifications affect that recovery. Comparatively less attention has been given to whether the fracture state existing before heating changes the apparent effectiveness of these recovery parameters. A high heating temperature may enhance binder softening, but it cannot fully compensate for severe interface separation. Similarly, extending curing time may provide additional opportunity for re-bonding, but its benefit is limited when the pre-heating fracture state has become too severe. Thus, microwave-assisted fracture energy recovery should be evaluated by considering both recovery parameters and pre-heating damage condition.

Most existing studies on asphalt mixture healing have focused on whether recovery can be achieved under a given heating method or material system, including mixtures containing metallic fibers, steel wool, carbon-based additives, steel slag, iron powder or microwave-responsive mineral components [9,10,11,12,13,14]. These studies provide important evidence for thermally activated recovery, but they primarily improve microwave susceptibility, heating efficiency or healing performance through material modification. Consequently, a higher measured recovery may reflect not only thermal activation but also differences in the mechanical state of the specimen before heating. In particular, the pre-heating condition is often prescribed as part of the test procedure rather than explicitly examined as a factor governing the subsequent recovery potential. For early-damaged mixtures, the key question is not simply whether a higher temperature or longer rest period improves recovery, but whether the mixture remains in a sufficiently favorable fracture state before heating. Therefore, a parameter-optimization framework is needed to quantify the factor importance and to determine the recovery condition under which early fracture damage can be most effectively restored.

Basalt fiber reinforcement provides a useful route for examining this damage-state regulation hypothesis. Basalt fibers have high tensile strength, thermal stability and crack-bridging capacity, and have been widely reported to improve rutting resistance, fatigue resistance and cracking resistance of asphalt mixtures [15,16,17,18,19]. Previous basalt-fiber studies have mainly focused on improving mechanical and crack resistance, whereas microwave-healing studies have mainly focused on thermal activation or healing enhancement. Although the influence of fiber reinforcement on microwave-assisted healing has also been investigated [10], whether the recovery advantage arises from a direct enhancement of the healing process or from the higher fracture resistance and consequently more favorable pre-heating state remains insufficiently distinguished. Under the same external load, a fiber-reinforced mixture with higher initial fracture resistance may correspond to a lower relative loading state than a control mixture. Consequently, it may retain higher fracture energy recovery potential even under the same microwave recovery condition. This provides the specific research gap addressed in the present study: the role of basalt fiber is examined not simply as a healing additive, but as a reinforcement component that may regulate the pre-heating fracture state and thereby influence subsequent recovery potential.

This study therefore investigates microwave-assisted fracture energy recovery in early-damaged asphalt mixtures from the perspective of damage-state regulation by basalt fiber reinforcement. A control asphalt mixture (CAM) and a basalt fiber-reinforced asphalt mixture (BFAM) were designed to provide different initial fracture-resistance levels. Semi-circular bending (SCB) fracture tests were conducted to characterize initial fracture resistance, and SCB damage–recovery tests were performed under three pre-heating damage conditions, three target surface temperatures and three curing times. An L9 orthogonal design was used to quantify the relative importance of these recovery factors, and the fracture energy recovery index was adopted to evaluate recovery effectiveness. The objectives of this study are to: (i) establish an SCB-based early-damage microwave recovery evaluation procedure; (ii) identify the dominant factors controlling fracture energy recovery; and (iii) clarify how basalt fiber reinforcement improves recovery potential by regulating the pre-heating damage condition before heating. The novelty of this study lies in separating the fracture-resistance contribution of basalt fiber reinforcement from the subsequent microwave-assisted recovery response and in evaluating their relationship through pre-heating damage condition control and a same-load comparison. The proposed recoverable damage window is therefore used as a conceptual framework to describe the range of pre-heating states associated with meaningful recovery, rather than as a quantitatively defined material damage variable.

## 2. Materials and Experimental Programme

### 2.1. Materials and Mixture Design

#### 2.1.1. Asphalt Binder

A commercially available SBS-modified asphalt binder with a performance grade of PG76-22 was used in this study. The penetration at 25 °C was 71 (0.1 mm), the softening point was 64 °C, the ductility at 5 °C was 48 cm, and the Brookfield viscosity at 135 °C was 1.75 Pa·s. The main properties of the asphalt binder satisfied the requirements of JTG F40.

#### 2.1.2. Aggregates

Basalt aggregates were used as the main coarse aggregates, while limestone aggregate was used for gradation adjustment. The basalt and limestone aggregates were produced by mechanical crushing and therefore exhibited irregular, angular particle shapes. The physical properties of the aggregates are summarized in Table 1. Their microwave heating response was evaluated to confirm that the mixtures could reach the target recovery surface temperatures.

#### 2.1.3. Mineral Filler

This study utilizes limestone as the primary mineral filler. The filler was clean, dry and free of agglomeration. Its relative density was 2.654, and its hydrophilic coefficient was 0.63, indicating good compatibility with the asphalt binder. The measured water content was 0.3%. The passing percentages of particles smaller than 0.6 mm, 0.15 mm and 0.075 mm were 100%, 98.5% and 82.5%, respectively, satisfying the gradation requirements for mineral filler in asphalt mixtures.

#### 2.1.4. Basalt Fibers

Chopped basalt fibers with a length of 6 mm were used as the reinforcing additive. The fibers had high tensile strength and good thermal resistance. Their main properties are listed in Table 2.

#### 2.1.5. Mixture Design

The asphalt mixture configuration evaluated herein was a dense-graded Superpave-13 mix, characterized by a nominal maximum aggregate size (NMAS) of 13.2 mm. The experimental design involved the preparation of two mixtures: a baseline control asphalt mixture and a modified mixture containing 0.3% basalt fibers by total weight of the asphalt mixture. The fiber content was selected according to the recommended value in the Technical Guidelines for Basalt Fiber Asphalt Pavement Construction (T/CHTS 10016-2019). The 0.3% fiber dosage was adopted as a representative dosage recommended by the specification rather than as an optimum dosage for microwave-assisted recovery. Optimization of fiber content was beyond the scope of this study. The two mixtures are denoted as CAM and BFAM, respectively. BFAM was included to provide a mixture with higher initial damage resistance, allowing the role of basalt fiber reinforcement in regulating the pre-heating damage state to be examined.

**Figure 1:**
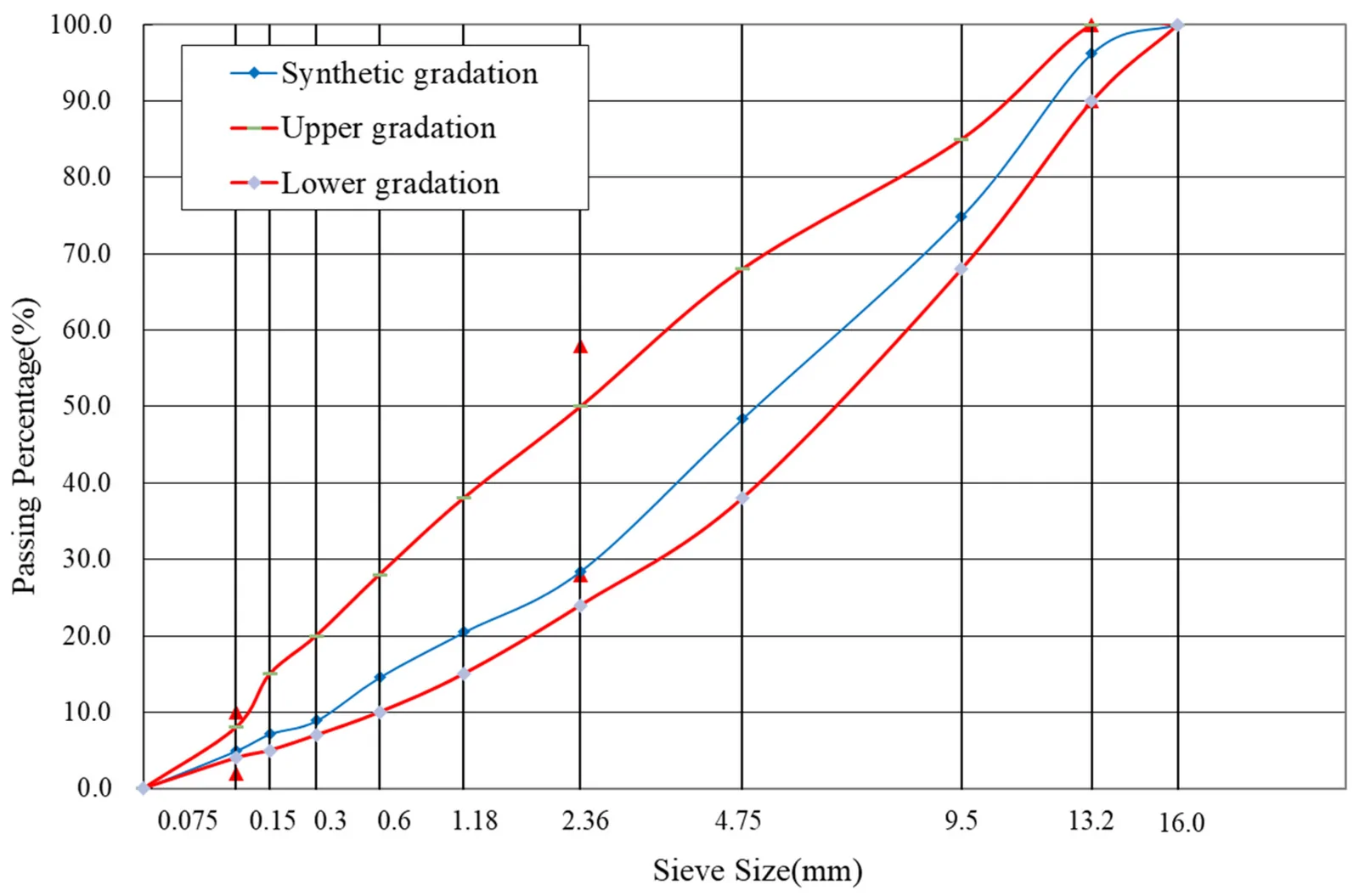
The designed gradation of Superpave-13 mixture.

The optimum asphalt–aggregate ratio was 4.7% for CAM and 4.9% for BFAM. The slightly higher asphalt–aggregate ratio of BFAM was adopted to account for the oil absorption and spatial reinforcement effect of basalt fibers. Both mixtures satisfied the Superpave volumetric design requirements. The designed gradation of the Superpave-13 mixture is shown in Figure 1. The target air void content of the gyratory-compacted specimens used for mixture design was approximately 4.0%.

### 2.2. Microwave Heating Feasibility Test

**Figure 2:**
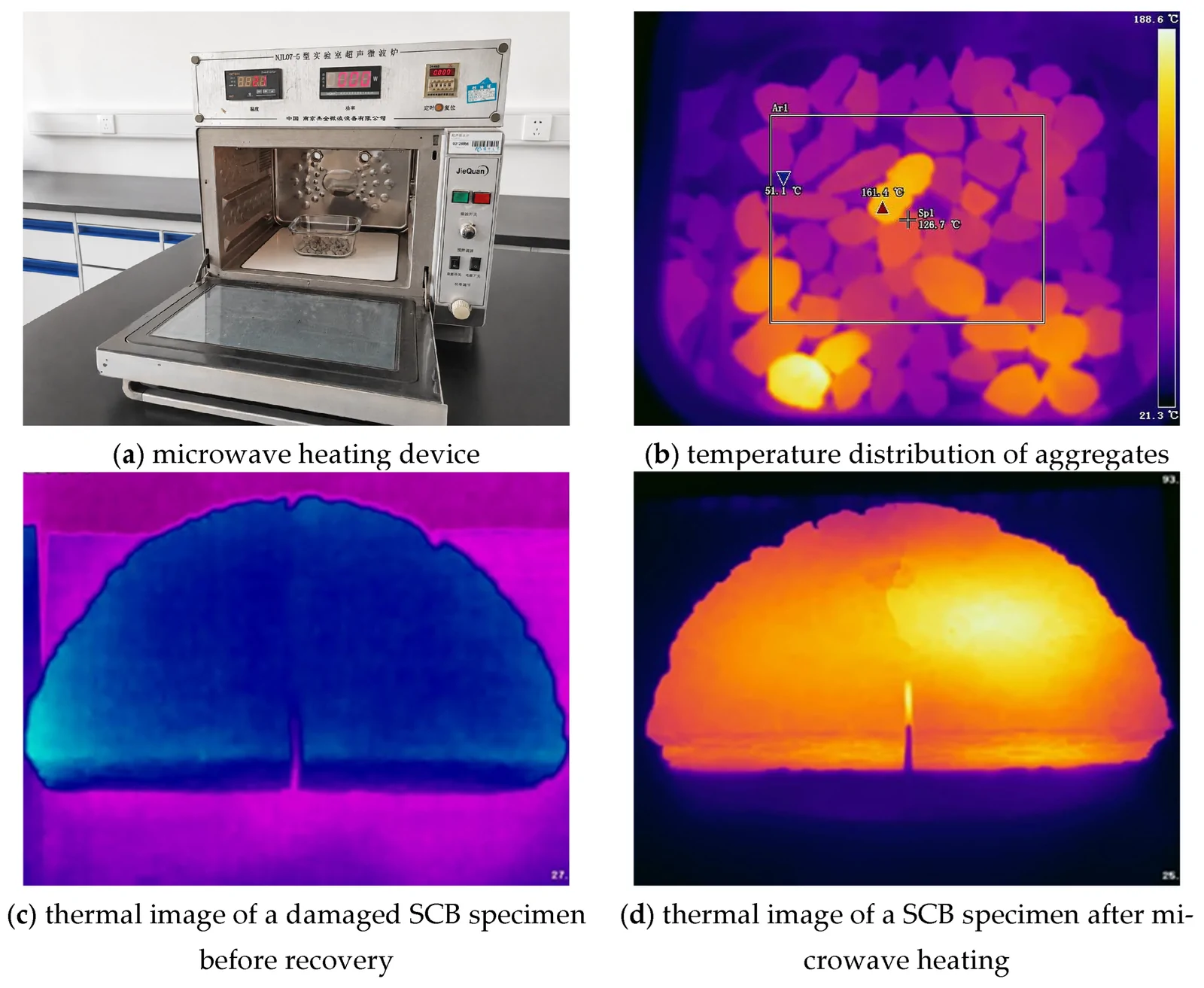
Microwave heating test setup and representative infrared thermal images.

Microwave heating tests were conducted to verify whether the aggregate–mixture system could reach the target surface temperatures required for the subsequent microwave-assisted fracture energy recovery tests, as shown in Figure 2. The tests were performed at both aggregate and mixture levels. For the aggregate-level test, three types of basalt aggregate and one type of limestone aggregate were heated in a microwave oven with a power of 800 W and a frequency of 2.45 GHz. The heating durations were 30, 60, 90, 120 and 150 s. After each heating stage, the aggregate temperature was measured using an infrared thermal imager (FLUKE Ti480U/CN).

Mixture-level microwave heating tests were then conducted using SCB specimens prepared with the selected aggregates. The specimens were heated for up to 180 s, and the surface temperature evolution was recorded using the infrared thermal imager. The purpose of this test was to determine whether the asphalt mixture specimens could reach the designed recovery temperatures within a practical microwave heating duration. No additional microwave absorbers, such as steel wool, steel fibers, carbon fibers, ferrite or carbon black, were added to the mixtures.

The average heating rate was defined as the average increase in the measured surface temperature per unit microwave-heating time and was calculated using Equation (1):

(1) $$v=\frac{T_{f}-T_{i}}{t}$$

where *v* is surface heating rate over the specified heating interval, *T_f_* is the final average surface temperature after microwave heating, *T_i_* is the initial average surface temperature, and *t* is the heating time.

To examine the possible reason for the difference in microwave responsiveness among aggregates, X-ray fluorescence spectroscopy (XRF-1800, Shimadzu Corporation, Kyoto, Japan) was used to determine the main oxide compositions of the aggregates. The Fe_2_O_3_ content was considered as a supporting indicator for explaining the aggregate heating response, rather than as the sole controlling factor. The infrared measurements represent the specimen surface temperature rather than the internal temperature. Because microwave heating may produce through-thickness temperature gradients, the internal temperature could differ from the measured surface temperature; this uncertainty was not directly quantified in the present feasibility test.

### 2.3. Pavement Performance Tests for Damage Resistance Verification

To verify the damage resistance of the designed asphalt mixtures, their road performances—encompassing high-temperature rutting resistance, low-temperature cracking resistance, fatigue behavior, and moisture stability—were characterized in compliance with the JTG 3410-2025 standard [21], using T0719, T0715, T0739, and T0709 & T0729, respectively.

High-temperature stability was evaluated by the wheel tracking test at 60 °C. Low-temperature cracking resistance was evaluated by the bending test at −10 °C. Fatigue resistance was evaluated by the four-point bending fatigue test at 15 °C under a controlled strain level of 450 με. To assess the resistance to moisture-induced damage, the residual Marshall stability and the splitting tensile strength ratio of the mixtures were evaluated. For each pavement performance test, triplicate specimens were evaluated for each asphalt mixture, with the arithmetic mean reported for subsequent analysis.

### 2.4. SCB Fracture Damage–Recovery Test and Orthogonal Design

SCB tests were used to evaluate the initial fracture resistance and microwave-assisted fracture energy recovery after controlled damage. Cylindrical specimens with a diameter of 150 mm were fabricated using a Superpave gyratory compactor (SGC) and subsequently sectioned into semi-circular geometries with a thickness of 50 ± 1 mm. A notch with a length of 15 mm was introduced at the center of the flat side edge to guide crack propagation.

The SCB fracture test was conducted at 25 °C with a vertical displacement rate of 50 mm/min. The load–displacement curve was continuously recorded during the test. Fracture work (W_f_), fracture energy (G_f_), post-peak slope (m), and flexibility index (FI) were calculated to characterize the fracture response, according to AASHTO T 393-22 [22] “Standard Method of Test for Determining the Fracture Potential of Asphalt Mixtures Using the Illinois Flexibility Index Test (I-FIT).”

The fracture work was calculated as the area under the load–displacement curve, as shown in Equation (2):

(2) $$W_{f}=\int P(u)du$$

Fracture energy and flexibility index were calculated using Equations (3) and (4), respectively:

(3) $$G_{f}=\frac{W_{f}}{A_{lig}}$$

(4) $$FI=\frac{G_{f}}{\left|m\right|}\times A$$

where *A_lig_* is the ligament area of the SCB specimen, *m* is the post-peak slope and *A* is a unit conversion factor.

For the recovery tests, three pre-heating damage conditions were established using the reference peak load (*P_max_*) obtained from the complete-fracture SCB tests of each mixture. For the two partial-damage conditions, specimens were loaded to 40% and 70% of the corresponding reference *P_max_*, respectively, and were then immediately unloaded. These loading levels were used as operational indices to generate different pre-heating fracture states and should not be interpreted as indicating that the physical fracture damage increased linearly with *P*/*P_max_*. For the complete-fracture condition, specimens were continuously loaded beyond *P_max_* through the post-peak stage until complete fracture occurred. This condition was included as a severe-damage boundary for comparison with the two partially damaged states.

**Figure 3:**
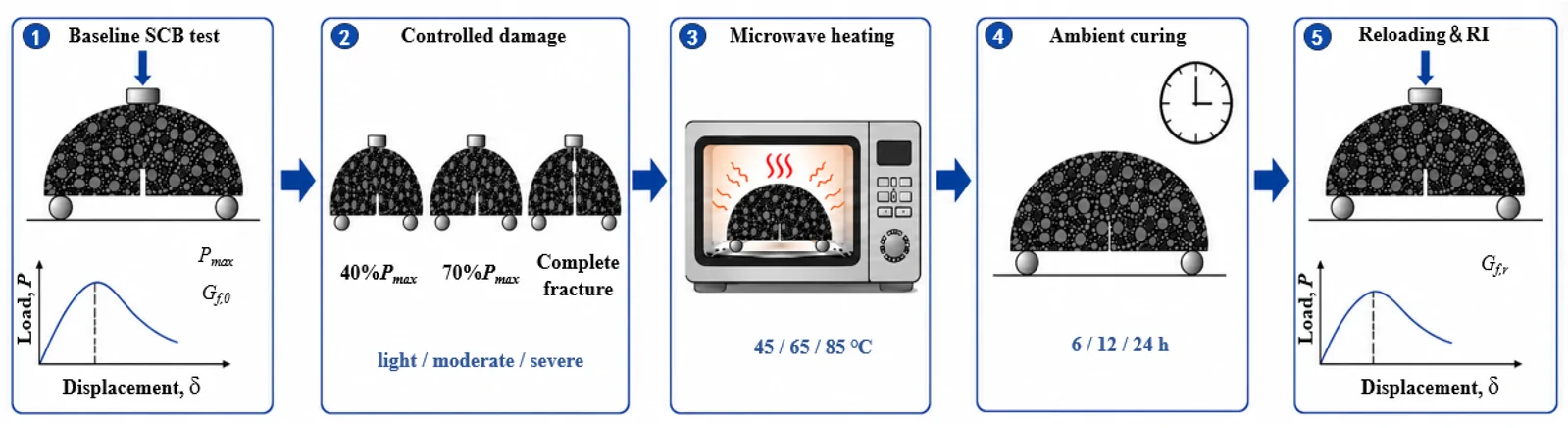
Schematic illustration of the SCB fracture damage and microwave-assisted recovery testing procedure.

After controlled damage, the SCB specimens were subsequently microwave-heated to target surface temperatures of 45, 65 and 85 °C. The average surface temperature was controlled within ±1 °C using infrared thermal monitoring. After microwave heating, the specimens were conditioned at ambient temperature to allow structural recovery for 6, 12 or 24 h, and then reloaded using the same SCB procedure. The overall SCB fracture damage–recovery procedure is illustrated in Figure 3.

The fracture energy recovery index *RI* was used to quantify the recovery level after microwave heating and curing, as defined in Equation (5):

(5) $$RI=\frac{G_{f,r}}{G_{f,0}}\times 100\%$$

where *G_f_*_, *r*_ is the fracture energy after controlled damage, microwave heating, curing and reloading, and *G_f_*_, 0_ is the mean initial fracture energy obtained from complete-fracture SCB specimens.

An L9 orthogonal array with three factors and three levels was adopted to evaluate the effects of pre-heating damage condition, surface temperature and curing time on fracture energy recovery. The factors and levels are summarized in Table 3. To guarantee statistical validity, tests were conducted in triplicate for each mixture configuration and testing condition. The orthogonal design was selected to evaluate the main effects of the three recovery parameters using nine treatment combinations rather than the 27 combinations required by a full 3^3^ factorial design. Because the L9 array was primarily designed for main-effect screening, interactions among the factors were not independently estimated.

Factor-level means and range values (*R*) were first calculated to describe the relative sensitivity of *RI* to each factor. In addition, a main-effects analysis of variance (ANOVA) was performed using the replicate-level *RI* data to statistically evaluate the influence of pre-heating damage condition, surface temperature, and curing time. The contribution ratio of each factor was calculated from its sum of squares relative to the total sum of squares. A significance level of *p* < 0.05 was adopted. For the complete-fracture SCB results, CAM and BFAM were compared using Welch’s independent-samples *t*-test because the comparison involved two independent mixture groups.

## 3. Results and Discussion

### 3.1. Microwave Heating Feasibility of the Asphalt Mixture System

**Figure 4:**
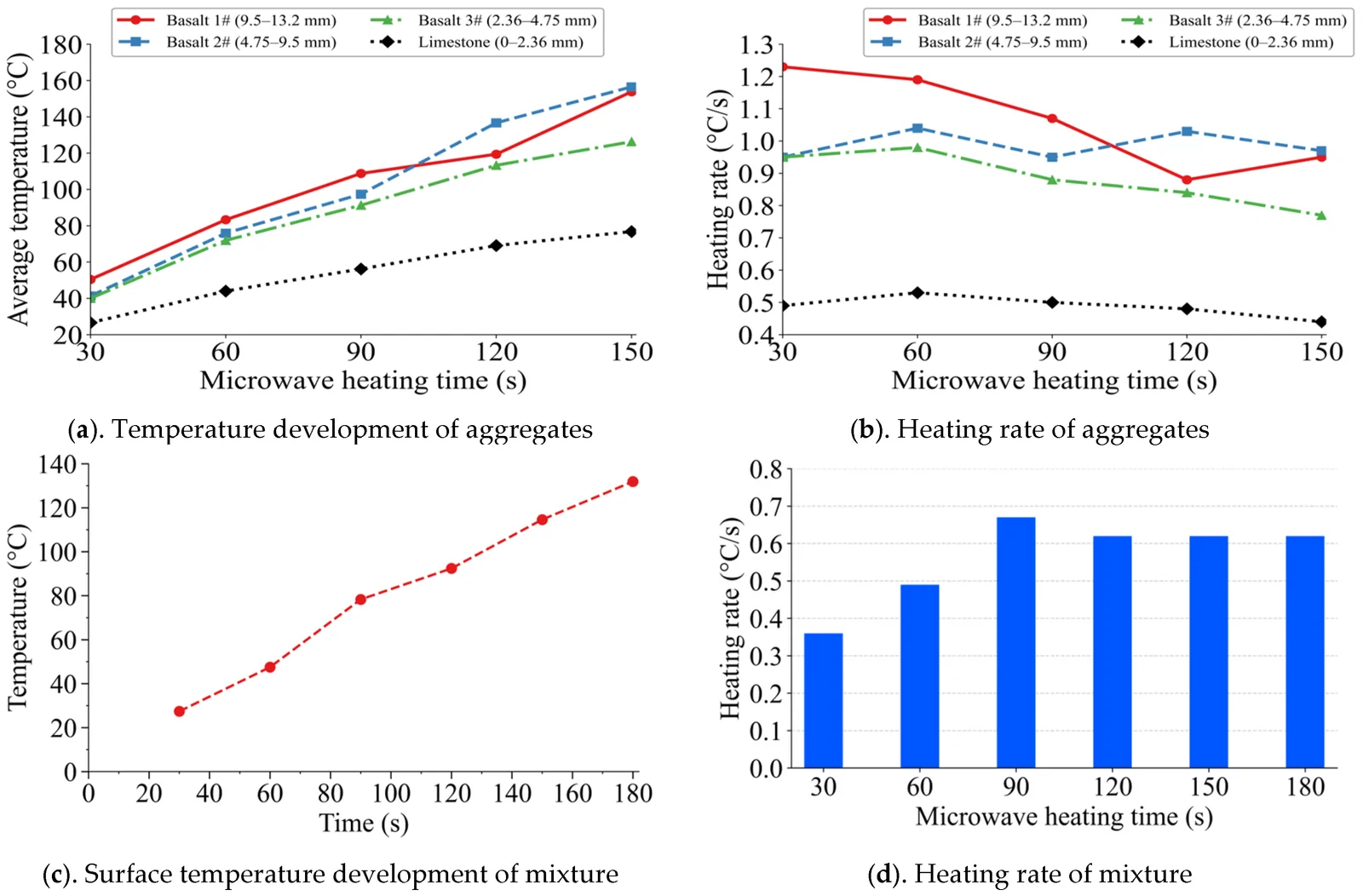
Microwave heating feasibility of aggregates and asphalt mixture specimens.

Microwave heating tests were conducted to verify whether the aggregate–mixture system could reach the target surface temperatures required for the subsequent fracture energy recovery tests. As shown in Figure 4a,b, the basalt aggregates exhibited a stronger microwave heating response than limestone. After 150 s of heating, the three basalt aggregates reached 126.3–156.5 °C, with average heating rates of 0.88–1.06 °C/s, whereas the limestone aggregate reached 76.8 °C with a heating rate of 0.49 °C/s. These results indicate that the selected basalt aggregates provided sufficient microwave responsiveness for the mixture system, consistent with previous studies on microwave-responsive aggregates in asphalt mixtures [13,14].

The different heating responses may be partly associated with aggregate mineral composition. The basalt aggregates contained higher Fe_2_O_3_ contents than limestone, which may contribute to their stronger microwave susceptibility. However, microwave heating behavior is also influenced by mineral composition, particle size, aggregate morphology, dielectric characteristics and heat dissipation conditions [11]. Therefore, Fe_2_O_3_ content was considered only as a supporting indicator rather than the sole controlling factor, and the detailed oxide compositions are provided in Table S1.

At the mixture level, the measured surface temperature increased from 27.4 °C at 30 s to 78.3 °C at 90 s and 131.9 °C at 180 s, confirming that the target surface temperatures of 45, 65 and 85 °C could be achieved within the selected microwave-heating duration without additional microwave absorbers. Thus, the aggregate–mixture system was considered suitable for the subsequent fracture energy recovery tests. Because these heating tests were intended as feasibility checks rather than replicated statistical comparisons, the heating curves were not used for inferential statistical analysis.

### 3.2. Initial Damage Resistance and Fracture Response of CAM and BFAM

**Figure 5:**
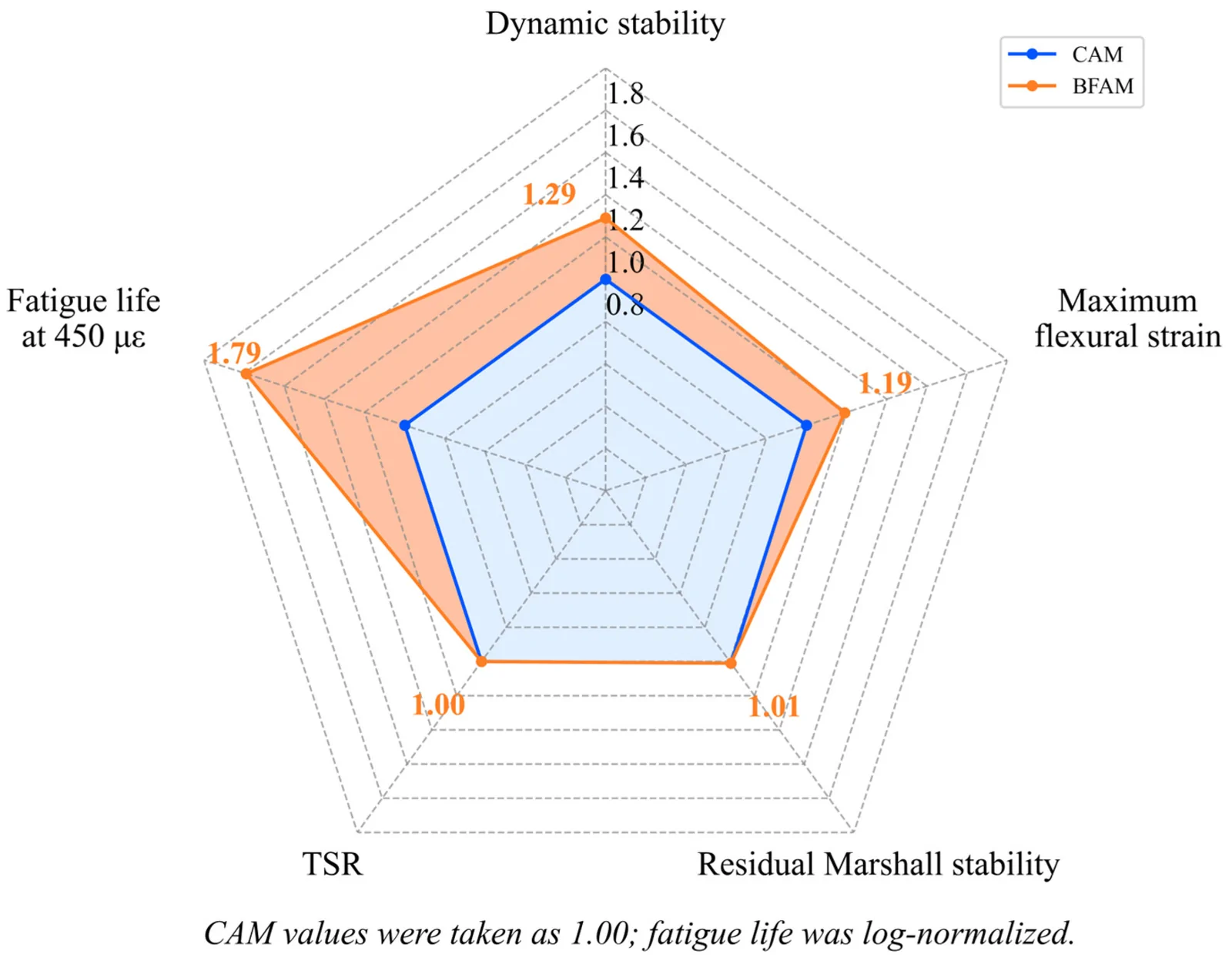
Normalized radar chart of pavement performance indices of CAM and BFAM.

The initial damage resistance of CAM and BFAM was first evaluated using conventional pavement performance indices, as shown in Figure 5. The purpose of this comparison was to verify whether basalt fiber reinforcement could provide a higher pre-damage resistance level before microwave-assisted fracture energy recovery. To compare indices with different units within the same framework, the CAM values were normalized to 1.00. Dynamic stability, maximum flexural strain, residual Marshall stability and tensile strength ratio were normalized using the direct ratio of BFAM to CAM. Because the fatigue life was much more sensitive to basalt fiber reinforcement than the other indices, its value at 450 με was transformed as 1 + log10 (*N_f_*_,_ *_BFAM_*/*N_f_*_,_ *_CAM_*) solely to avoid visual dominance in the radar chart. This author-defined transformation was used only for visualization and was not used in the statistical analysis or quantitative interpretation.

As shown in Figure 5, basalt fiber reinforcement improved the rutting resistance, low-temperature deformation capacity and fatigue damage resistance of the asphalt mixture, while its effect on moisture stability was limited. The normalized values of dynamic stability, maximum flexural strain and fatigue life increased to 1.29, 1.19 and 1.79, respectively. Specifically, the dynamic stability increased from 6015 cycles/mm for CAM to 7744 cycles/mm for BFAM, corresponding to an improvement of 28.7%. The maximum flexural strain increased from 3152 με to 3757 με, and the fatigue life at 450 με increased from 53.42 × 10^4^ cycles to 328.12 × 10^4^ cycles. In contrast, the residual Marshall stability increased only slightly from 94.06% to 95.10%, and the tensile strength ratio changed marginally from 88.95% to 89.12%. This limited improvement may be attributed to the predominantly mechanical reinforcement role of basalt fibers through crack bridging and stress redistribution, whereas moisture stability is more closely related to asphalt–aggregate interfacial adhesion and moisture-induced debonding. Since the fibers were not introduced as anti-stripping agents or interfacial adhesion modifiers, their influence on these moisture-stability indices remained limited. These results indicate that basalt fibers mainly enhanced the resistance of the mixture to deformation and repeated-load damage, rather than substantially improving moisture stability [23].

**Figure 6:**
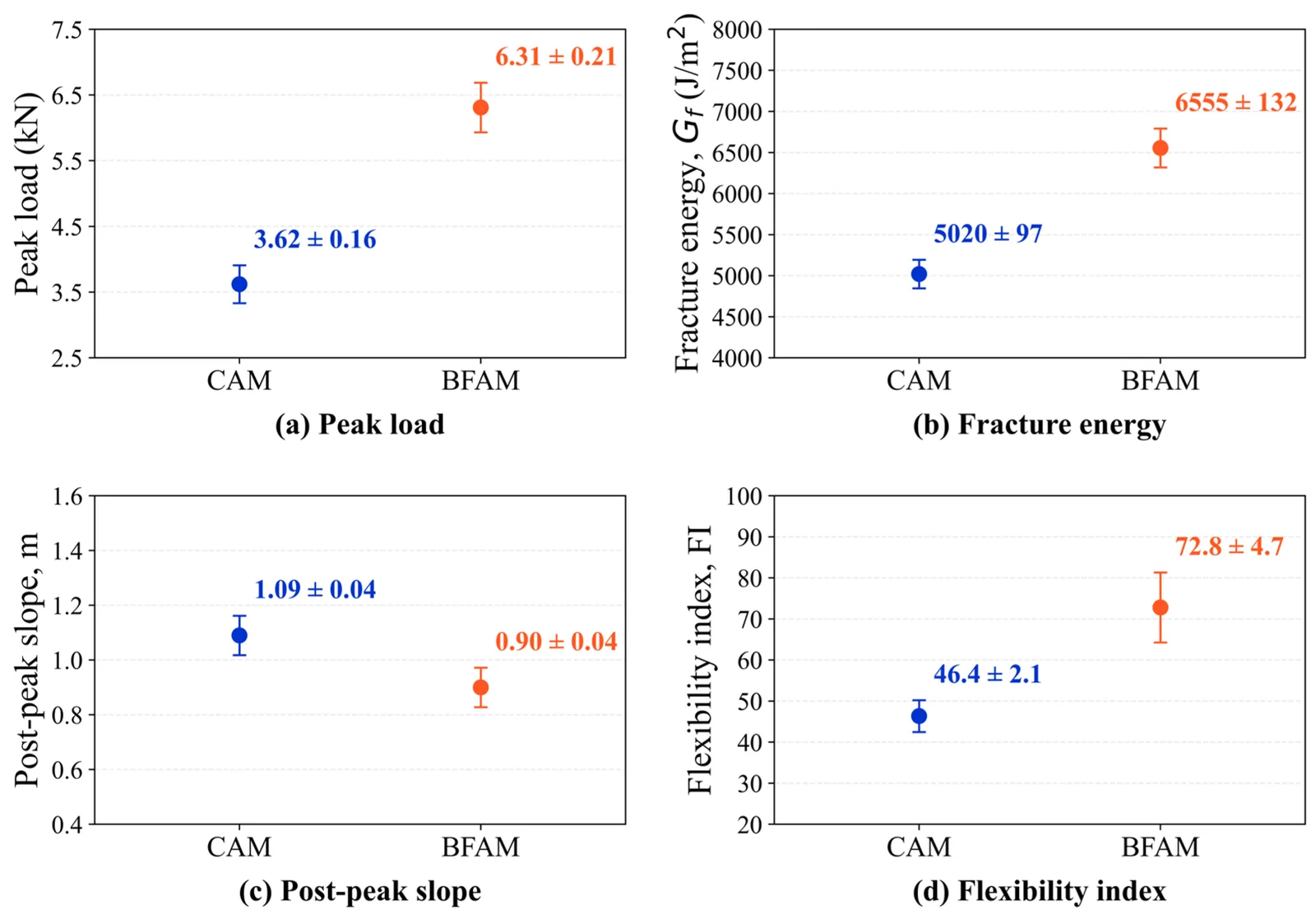
SCB fracture resistance of CAM and BFAM.

The SCB fracture test results provide a more direct evaluation of initial fracture damage resistance, as shown in Figure 6. The peak load increased from 3.62 kN for CAM to 6.31 kN for BFAM, while *G_f_* increased from 5020 J/m^2^ to 6555 J/m^2^ and *FI* increased from 46.4 to 72.8. Welch’s independent-samples *t*-tests further confirmed significant differences between CAM and BFAM in peak load, fracture energy, post-peak slope and flexibility index (all *p* < 0.001). These results indicate that BFAM required more energy for crack development and showed higher tolerance to crack propagation.

Overall, Figure 5 and Figure 6 demonstrate that basalt fiber reinforcement improved the initial damage resistance of the asphalt mixture, especially in terms of fatigue resistance and SCB fracture response. This improvement can be attributed to fiber bridging, stress redistribution and crack propagation restraint, which helped delay crack initiation and slow crack growth [24,25,26,27]. More importantly, these results provide the basis for interpreting the subsequent recovery behavior from the viewpoint of damage-state regulation. Under the same external load, BFAM is expected to remain at a lower relative loading state than CAM and therefore retain a more favorable pre-heating state before microwave heating.

### 3.3. Microwave-Assisted Fracture Energy Recovery Under L9 Conditions

**Figure 7:**
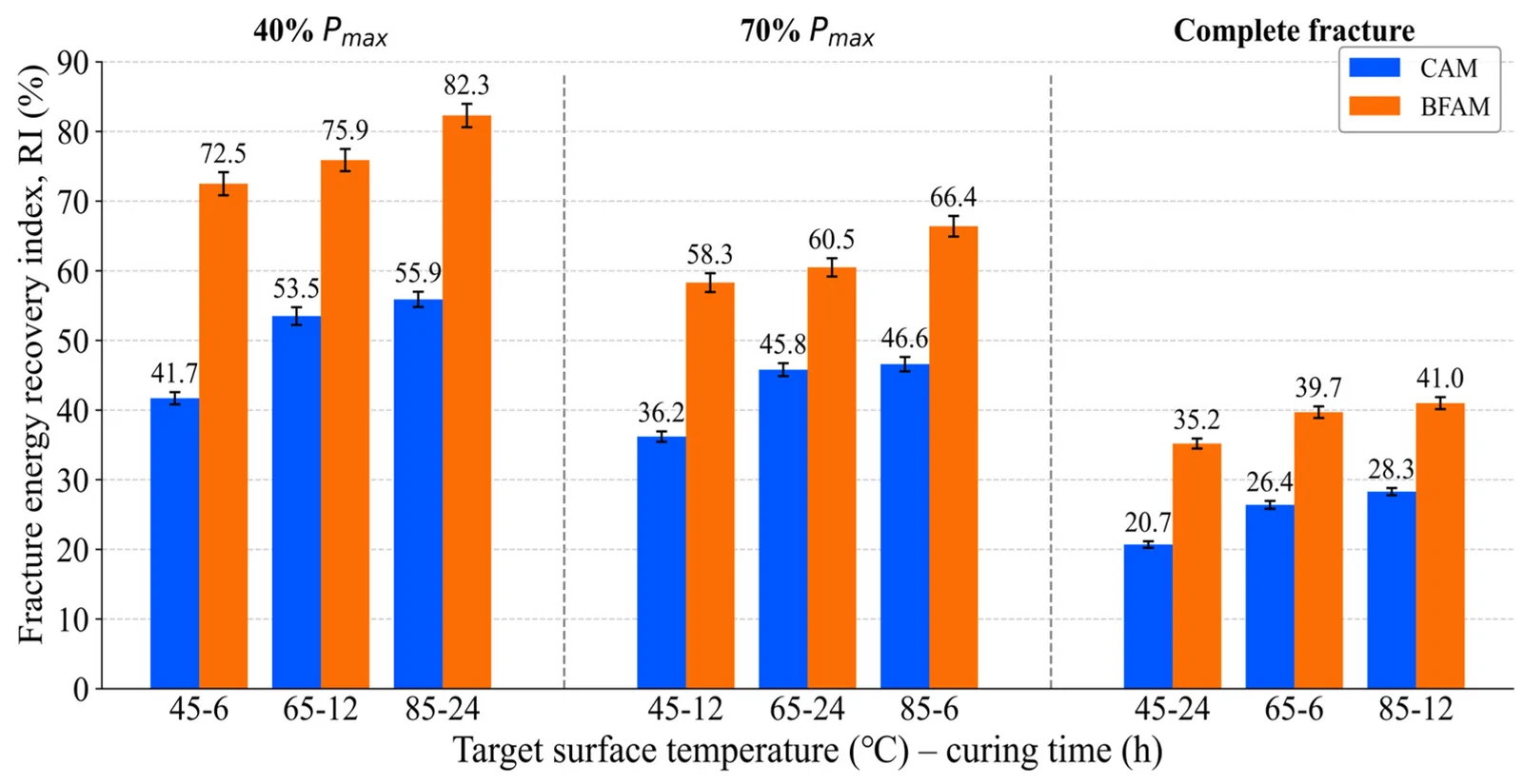
Comparison of fracture energy recovery index *RI* of CAM and BFAM under different conditions.

Microwave-assisted fracture energy recovery was evaluated using the recovery index *RI* calculated from three independent specimens for each L9 condition. The mean *RI* values and corresponding standard deviations of CAM and BFAM are shown in Figure 7.

Overall, BFAM exhibited higher *RI* values than CAM under all investigated recovery conditions. For CAM, the mean *RI* values ranged from 20.7% to 55.9%, whereas that of BFAM ranged from 35.2% to 82.3%. The consistently higher recovery levels of BFAM suggest that basalt fiber reinforcement helped the mixture retain a more favorable pre-heating fracture state. Similar fiber-bridging and crack-resistance mechanisms have also been reported for basalt fiber-reinforced asphalt mixtures in recent fracture studies [28]. However, this difference should not be interpreted as direct evidence that basalt fibers intrinsically enhanced binder healing; the role of fiber reinforcement is further examined through the same-load comparison in Section 3.5.

The pre-heating condition had a pronounced influence on fracture energy recovery. Under the 40% *P_max_* condition, the *RI* values were 41.7–55.9% for CAM and 72.5–82.3% for BFAM. Under the complete-fracture condition, *RI* further decreased to 20.7–28.3% for CAM and 35.2–41.0% for BFAM. Thus, progressively more severe pre-heating fracture conditions were associated with a marked reduction in recoverable fracture energy.

Among the L9 conditions, the highest recovery levels for both mixtures were obtained under the 40% *P_max_* condition, with surface temperature of 85 °C and a curing time of 24 h. The corresponding *RI* values were 55.9% for CAM and 82.3% for BFAM. These results show that the pre-heating fracture condition exerted a stronger apparent influence on recovery than changes in surface temperature or curing time. Because the L9 results combine the effects of all three factors, their relative contributions cannot be determined from Figure 7 alone. The factor-level effects and their statistical significance are therefore evaluated further using range analysis and ANOVA in Section 3.4.

### 3.4. Factor Importance and Recovery-Parameter Optimization

To quantify the effects of the recovery parameters, factor-level response and range analyses were first performed using the L9 orthogonal test results. For each factor, the *RI* values corresponding to the same level were averaged to obtain the factor-level mean. The range value *R* was calculated as the difference between the maximum and minimum factor-level means, as expressed in Equation (6):

(6) $$R=\max(RI_{1},RI_{2},RI_{3})-\min(RI_{1},RI_{2},RI_{3})$$

**Figure 8:**
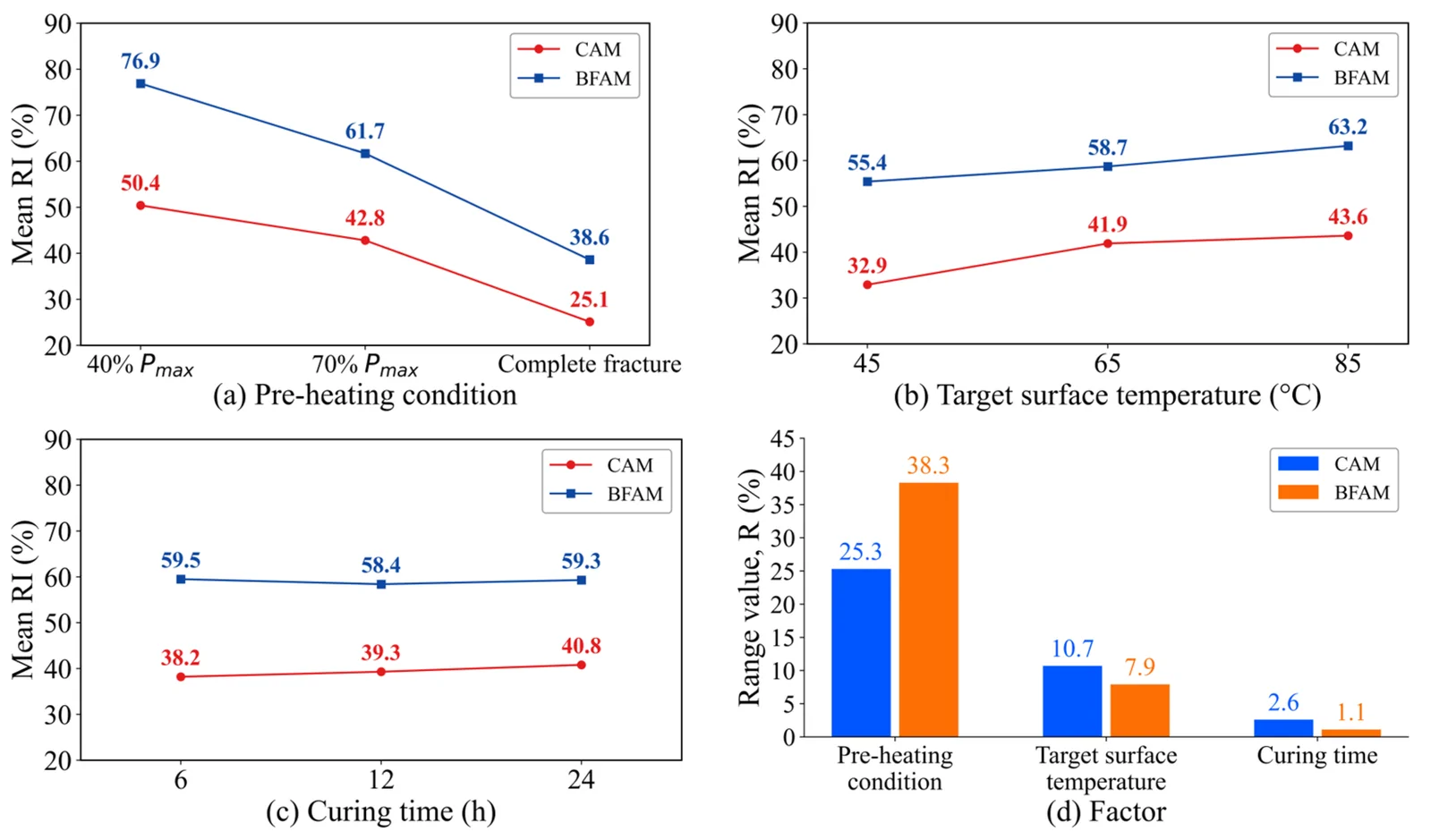
Factor-level means and range analysis of fracture energy recovery index *RI*.

A larger *R* value indicates a greater sensitivity of fracture energy recovery to that factor. The factor-level responses and range values are presented in Figure 8.

As shown in Figure 8a, pre-heating damage condition produced the largest variation in *RI*. For CAM, the average *RI* decreased from 50.4% under the 40% *P_max_* condition to 42.8% under the 70% *P_max_* condition and further to 25.1% after complete fracture. For BFAM, the corresponding values were 76.9%, 61.7%, and 38.6%, respectively. This trend indicates that the pre-heating damage state strongly controlled the recoverable fracture energy.

Surface temperature showed the second strongest influence on *RI*, as shown in Figure 8b. The average *RI* increased from 32.9% to 43.6% for CAM and from 55.4% to 63.2% for BFAM, when surface temperature increased from 45 °C to 85 °C. These results suggest that increasing the surface temperature was beneficial for fracture energy recovery, although its effect was smaller than that of the pre-heating damage condition.

In contrast, curing time produced only small changes in the factor-level means. As shown in Figure 8c, for CAM, the average *RI* increased from 38.2% at 6 h to 40.8% at 24 h, whereas the BFAM values remained within a narrow range of 58.4–59.5%. This indicates that extending the ambient curing time from 6 to 24 h provided limited additional recovery under the present test conditions.

The range analysis in Figure 8d further confirms the relative importance of the three factors. For CAM, the *R* values of pre-heating damage condition, surface temperature and curing time were 25.3, 10.7 and 2.6, respectively. For BFAM, the corresponding values were 38.3, 7.9 and 1.1. The resulting *R* values under the pre-heating damage condition were much larger than those of the other factors.

A main-effects ANOVA was further conducted using the *RI* data to evaluate the main-effect contributions within the adopted L9 experimental framework, as shown in Table 4. For CAM, the pre-heating damage condition accounted for 82.3% of the total variation in *RI*, followed by surface temperature at 16.2% and curing time at 0.80%. The effects of pre-heating damage condition and surface temperature were highly significant (*p* < 0.001). Curing time was also statistically significant for CAM (*p* < 0.001); however, its main-effect contribution to the overall variation was less than 1%, indicating limited practical influence within the investigated range.

A similar but more pronounced trend was obtained for BFAM. The main-effect contribution of pre-heating damage condition accounted for 95.2% of the total *RI* variation, while heating temperature contributed 4.0%. Both effects were statistically significant (*p* < 0.001). Curing time contributed only 0.09% and was not statistically significant (*p* = 0.307). Thus, both the range analysis and ANOVA consistently identified the following main-effect ranking: pre-heating damage condition > surface temperature > curing time.

Based on the factor-level responses, the combination producing the highest recovery within the investigated parameter space was the 40% *P_max_* pre-heating damage condition, a surface temperature of 85 °C, and a curing time of 24 h. Nevertheless, the statistical results show that preserving a less advanced pre-heating fracture state is considerably more influential than extending the curing period. The L9 design was used primarily for main-effect screening; therefore, possible interactions among the three factors were not independently quantified. Accordingly, the reported contribution ratios and factor ranking should be interpreted as main-effect results within the present L9 experimental framework, rather than as a complete decomposition including interaction effects.

### 3.5. Damage-State Regulation by Basalt Fiber Reinforcement Under a Comparable Load

The results in Section 3.3 and Section 3.4 showed that BFAM exhibited higher *RI* values than CAM and that the pre-heating damage condition was the dominant factor controlling fracture energy recovery. Since CAM and BFAM exhibited substantially different peak loads, comparison only at the same *P_max_* level does not fully reflect their response under a comparable external load. A same-load comparison was therefore introduced to further examine the damage-state regulation effect of basalt fiber reinforcement.

**Figure 9:**
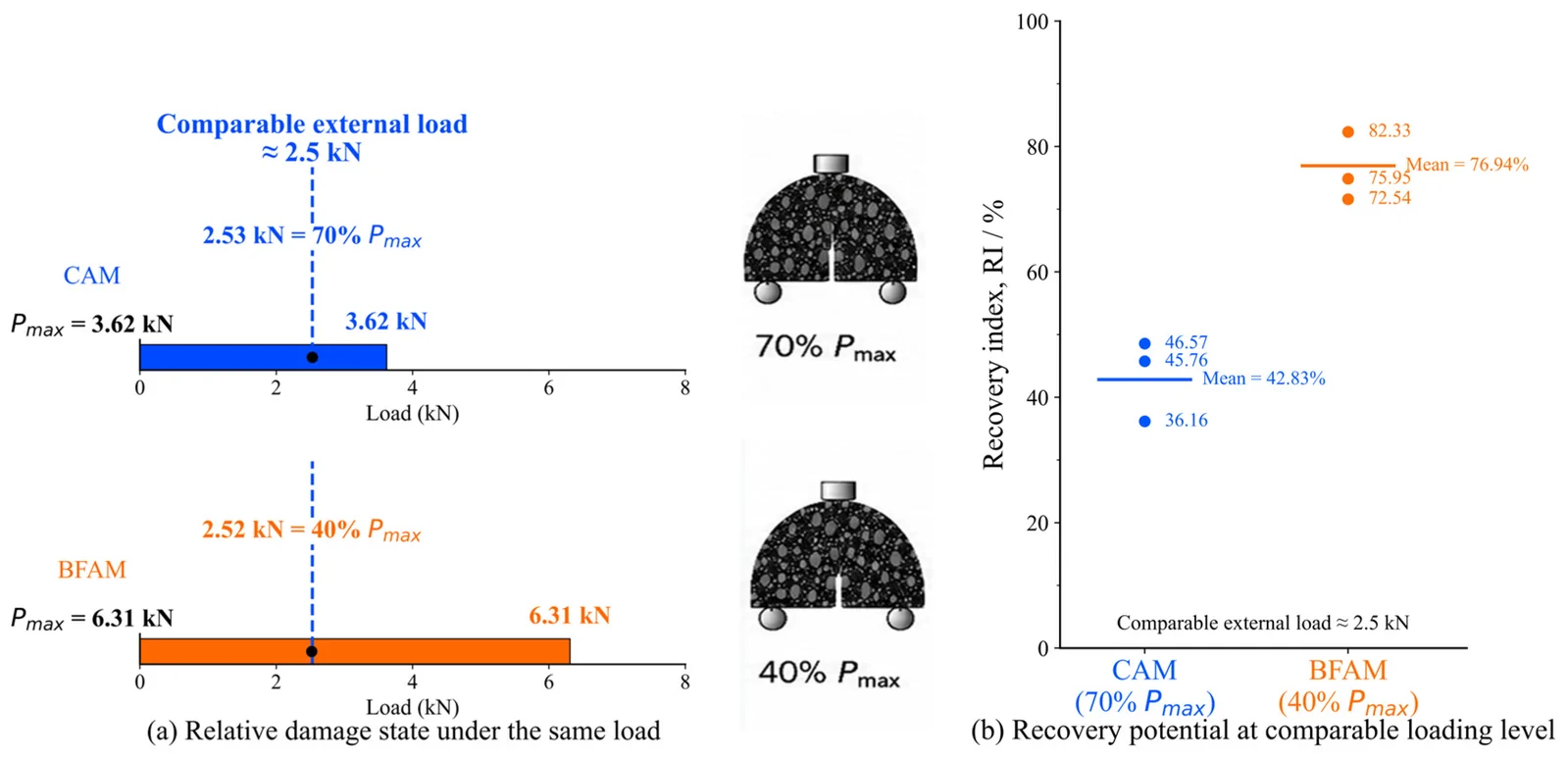
Recovery potential of CAM and BFAM under a comparable external load.

As shown in Figure 9a, according to the complete SCB fracture results, the peak load was 3.62 kN for CAM and 6.31 kN for BFAM. A load of approximately 2.5 kN corresponded to 70% *P_max_* for CAM but only 40% *P_max_* for BFAM. These two pre-heating damage conditions were produced under nearly the same external load, but they represented different relative loading states in the two mixtures. The corresponding recovery results are compared in Figure 9b. For CAM at 70% *P_max_* condition, the *RI* values for the three L9 combinations were 36.16%, 45.76%, and 46.57%, giving an overall mean of 42.83%. For BFAM at 40% *P_max_* condition, the *RI* values were 72.54%, 75.95% and 82.33%, with an average value of approximately 76.94%. Thus, BFAM retained a higher recovery potential across all selected recovery conditions, rather than only under the optimal condition.

This same-load comparison indicates that the higher *RI* of BFAM was mainly associated with its lower relative loading state before microwave heating. This more favorable pre-heating state was associated with the higher recovery potential of BFAM. From a mechanistic perspective, the higher fracture resistance may help preserve conditions that are more favorable for subsequent microwave-assisted recovery [29]. Therefore, basalt fiber reinforcement can be interpreted as a damage-state regulation strategy that may broaden the proposed recoverable damage window. Although a similar tendency may be expected at other common load levels because of the higher fracture resistance of BFAM, the magnitude of the loading-state difference and the corresponding recovery advantage cannot be generalized without additional recovery tests at multiple common loading levels.

### 3.6. Parameter-Controlled Recovery Mechanism and Limitations

**Figure 10:**
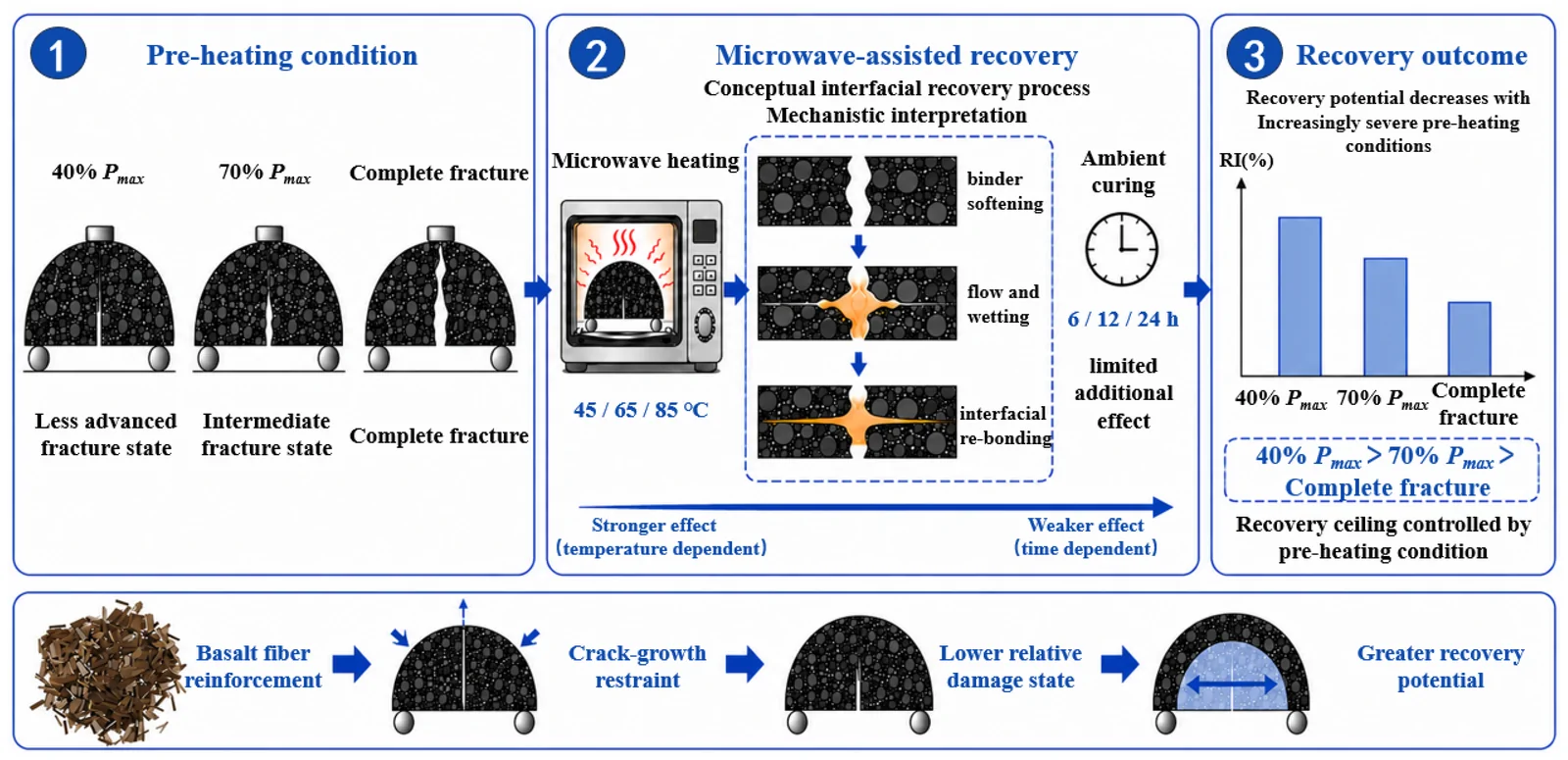
Proposed conceptual framework for damage-state-regulated microwave-assisted fracture energy recovery.

Based on the experimental results, a conceptual framework for damage-state-regulated microwave-assisted fracture energy recovery is proposed in Figure 10. The framework distinguishes the directly observed experimental responses from the mechanistic interpretation of the recovery process. The L9 results showed that recovery decreased systematically as the pre-heating damage condition progressed from 40% *P_max_* to complete fracture, while the ANOVA further identified the pre-heating condition as the dominant main effect. The three components of the proposed framework are respectively supported by the pre-heating-condition effects identified in Section 3.3 and Section 3.4, the temperature and curing responses obtained from the L9 analysis, and the fracture-resistance and same-load comparisons presented in Section 3.2 and Section 3.5.

Microwave-assisted fracture energy recovery can be interpreted as a damage-state-controlled process. The pre-heating damage condition is interpreted as influencing the subsequent recovery potential through the extent of fracture development before treatment. Under the 40% *P_max_* condition, the fracture state is less advanced and the opposing fracture surfaces are expected to retain greater proximity, which may provide more favorable conditions for binder flow, wetting, and interfacial re-bonding during microwave-assisted recovery. With increasing pre-heating severity, crack opening and interface separation are expected to become more pronounced, reducing the effective contact area and limiting the recovery level. Under the complete fracture condition, extensive interface separation is expected to restrict the ability of heating and curing to restore the original fracture energy. Since crack morphology was not directly characterized by CT, microscopy or related techniques, the interfacial evolution illustrated in Figure 10 should be regarded as a mechanistic interpretation rather than direct experimental observation.

Target surface temperature represents the main external thermal-control parameter in the present experimental framework. From a mechanistic perspective, increasing the target surface temperature is expected to promote binder softening, reduce viscosity, and facilitate binder flow and wetting around the fracture interface, which may contribute to interfacial re-bonding and fracture energy recovery. However, the effect of surface temperature depends on the pre-heating damage condition. If extensive interface separation has occurred, increasing the temperature alone would not be expected to fully compensate for the loss of interface contact and material continuity. This agrees with the factor-level and ANOVA results, where the pre-heating damage condition showed a stronger influence than surface temperature. Recent investigations also indicate that temperature elevation enhances healing efficiency but cannot fully restore severely damaged interfaces [30].

Curing time had a weaker effect within the investigated range of 6–24 h. The limited curing-time effect suggests that a substantial part of the recovery may occur during or shortly after microwave heating, when the binder is thermally activated and may remain capable of interfacial wetting and redistribution. Extending ambient curing time provided only limited additional recovery, especially for BFAM. Although curing time was statistically significant for CAM, its contribution to the total *RI* variation was below 1%; for BFAM, its effect was not statistically significant. Thus, the recovery process should not be regarded as a simple time-dependent healing process. A favorable pre-heating damage condition and sufficient thermal activation are more critical than merely prolonging the curing period. This observation is consistent with recent findings emphasizing the important role of temperature-driven binder diffusion in asphalt healing [31].

Basalt fiber reinforcement modifies the recovery process by regulating the damage state before microwave treatment. As shown by the initial fracture tests and same-load comparison, BFAM had higher fracture resistance and reached a lower relative loading state under the same external load. Mechanistically, the fiber network may restrain crack growth, redistribute local stress, and reduce abrupt crack opening, thereby helping to preserve a more favorable pre-heating state [29]. These results therefore suggest that the contribution of basalt fiber reinforcement mainly lies in preserving a favorable pre-heating state and thereby potentially broadening the proposed recoverable damage window, rather than directly enhancing binder recovery.

The proposed framework also provides implications for early maintenance and material design. The present results suggest that microwave-assisted recovery may be more effective when applied before extensive fracture development, while the mixture remains within the proposed recoverable damage window. For mixtures with higher initial damage resistance, such as BFAM, the same traffic-induced load may correspond to a lower relative loading state, allowing greater recovery potential to be retained. Thus, within the present experimental framework, improving damage resistance and controlling the pre-heating damage condition are more effective than relying only on higher surface temperature or longer curing time. The optimum combination identified in this study should therefore be regarded as a laboratory optimum within the investigated parameter range rather than a direct field-treatment prescription.

Several limitations should be acknowledged. The recovery behavior was evaluated using laboratory SCB fracture energy tests, and field-scale healing performance was not directly assessed. In addition, only surface temperature was monitored during microwave heating, while internal temperature gradients require further investigation. Only one SBS-modified binder, one Superpave-13 gradation and one basalt fiber dosage were investigated; therefore, the proposed framework should be interpreted within the material system examined in this study. Moreover, the proposed recoverable damage window remains a conceptual framework; its boundaries were not quantitatively determined from direct crack-morphology measurements and require further validation under broader material systems and loading–recovery conditions. The long-term durability, repeated recovery performance, and field-scale heating uniformity should also be examined in future work.

## 4. Conclusions

This study investigated microwave-assisted fracture energy recovery in asphalt mixtures, from the perspective of damage-state regulation by basalt fiber reinforcement. The following conclusions can be drawn:

(1) Basalt fiber reinforcement significantly improved the initial fracture resistance of the asphalt mixture. Compared with CAM, BFAM exhibited higher peak load, fracture energy, and flexibility index, with all differences being statistically significant (*p* < 0.001).

(2) Fracture energy recovery decreased markedly as the pre-heating damage condition progressed from 40% *P_max_* to 70% *P_max_* and complete fracture. The *RI* ranged from 20.7% to 55.9% for CAM and from 35.2% to 82.3% for BFAM, showing that a less advanced pre-heating fracture state retained greater recovery potential.

(3) Main-effects ANOVA identified the pre-heating damage condition as the dominant factor within the adopted L9 framework, with main-effect contributions of 82.3% and 95.2% for CAM and BFAM, respectively. Surface temperature had a secondary effect, whereas curing time made only a minor contribution. The highest recovery within the investigated range occurred at 40% *P_max_* condition, a surface temperature of 85 °C, and a curing time of 24 h.

(4) The same-load comparison supported the damage-state regulation role of basalt fiber reinforcement. At approximately 2.5 kN, CAM reached the 70% *P_max_* condition, whereas BFAM remained at the 40% *P_max_* condition, with corresponding mean *RI* values of 42.8% and 76.9%. This suggests that fiber reinforcement enhances recovery potential mainly by delaying progression toward complete fracture rather than by directly enhancing binder recovery.

(5) From an engineering perspective, improving fracture resistance and applying microwave-assisted treatment before extensive fracture development are more important for enhancing recovery potential. The identified optimum is limited to the investigated material system, and the proposed recoverable damage window requires further validation.

## Figures and Tables

**Table 1:** Physical properties of aggregates.

Type	Particle Size /mm	Apparent Relative Density	Bulk Relative Density	Flaky and Elongated Particles Content/%
Basalt	9.5–13.2	2.968	2.908	3.6
	4.75–9.5	2.953	2.902	4.3
	2.36–4.75	2.946	2.853	/
Limestone	0–2.36	2.700	N/A	/

**Table 2:** Properties of chopped basalt fibers.

Properties	Results	Requirement in T/CHTS 10016-2019 [20]
Elongation at break/%	2.71	≤3.1
Breaking strength/MPa	2218	≥1200
Oil absorption rate/%	52	≥50
Heat resistance, breaking strength retention rate/%	93	≥85
Alkali resistance, breaking strength retention rate/%	89	≥75

**Table 3:** Orthogonal design of SCB fracture energy recovery tests.

Run	Pre-Heating Damage Condition	Surface Temperature /°C	Curing Time /h
1	40% *P_max_*	45	6
2	40% *P_max_*	65	12
3	40% *P_max_*	85	24
4	70% *P_max_*	45	12
5	70% *P_max_*	65	24
6	70% *P_max_*	85	6
7	Complete fracture	45	24
8	Complete fracture	65	6
9	Complete fracture	85	12

**Table 4:** Main-effects ANOVA of the fracture energy recovery index.

Mixture	Factor	d_f_	F	*p*-Value	Main-Effect Contribution/%
CAM	Pre-heating condition	2	1142.45	<0.001	82.27
	Surface temperature	2	224.99	<0.001	16.20
	Curing time	2	11.16	<0.001	0.80
BFAM	Pre-heating condition	2	1290.91	<0.001	95.19
	Surface temperature	2	53.94	<0.001	3.98
	Curing time	2	1.25	0.307	0.09

## Data Availability

The original contributions presented in this study are included in the article. Further inquiries can be directed to the corresponding author.

## Supplementary Materials

The following supporting information can be downloaded at: https://www.mdpi.com/article/10.3390/ma19163536/s1, Table S1: Chemical composition of aggregates used for microwave heating analysis.
