# Supplementary material for: Optimization of Microwave-Assisted Fracture Energy Recovery in Early-Damaged Asphalt Mixtures: Damage-State Regulation by Basalt Fiber Reinforcement
Source: Materials (Basel). 2026 Aug 20;19(16):3536. doi: 10.3390/ma19163536 (PMC13514388; doi:10.3390/ma19163536)
Supplement: Supplementary file 1 [file materials-19-03536-s001.zip › materials-4463987-Supplementary Table S1.pdf]

Table S1. Chemical composition of aggregates used for microwave heating analysis.

Sample	SiO_2	MgO	Al_2O_3	Fe_2O_3	CaO	Na_2O	Others
Basalt 1#	47.41%	10.83%	13.08%	14.22%	7.75%	3.02%	3.69%
Basalt 2#	53.20%	11.08%	10.53%	10.33%	8.27%	2.62%	4.19%
Basalt 3#	52.91%	9.28%	12.88%	10.11%	7.33%	3.16%	4.11%
Limestone 4#	17.61%	3.31%	4.81%	2.87%	69.07%	0.00%	2.33%
